# Tuberous Sclerosis Complex with rare associated findings in the gastrointestinal system: a case report and review of the literature

**DOI:** 10.1186/s12876-020-01481-y

**Published:** 2020-11-23

**Authors:** Larissa Brussa Reis, Daniele Konzen, Cristina Brinckmann Oliveira Netto, Pedro Moacir Braghirolli Braghini, Gabriel Prolla, Patricia Ashton-Prolla

**Affiliations:** 1 Laboratório de Medicina Genômica - Centro de Pesquisa Experimental - Hospital de Clinicas de Porto Alegre (HCPA), Porto Alegre, Rio Grande do Sul Brazil; 2 Programa de Pós-graduação em Genética e Biologia Molecular, Universidade Federal do Rio Grande do Sul (UFRGS), Porto Alegre, Rio Grande do Sul Brazil; 3 Hospital Mãe de Deus, Porto Alegre, Rio Grande do Sul Brazil; 4 Hospital São Lucas, Escola de Medicina da Pontifícia Católica do Rio Grande do Sul (PUCRS), Porto Alegre, Rio Grande do Sul Brazil; 5 Serviço de Genética Médica, Hospital de Clinicas de Porto Alegre (HCPA), Rua Ramiro Barcelos 2350, Porto Alegre, RS CEP: 90035-903 Brazil; 6 Hospital São Vicente de Paulo, Passo Fundo, Rio Grande do Sul Brazil

**Keywords:** Tuberous sclerosis complex, Adenomatous colonic, Rectal polyposis, Pancreatic neuroendocrine tumor, Case report

## Abstract

**Background:**

Tuberous Sclerosis Complex (TSC) is a complex and heterogeneous genetic disease that has well-established clinical diagnostic criteria. These criteria do not include gastrointestinal tumors.

**Case presentation:**

We report a 45-year-old patient with a clinical and molecular diagnosis of TSC and a family history of cancer, presenting two rare associated findings: gastrointestinal polyposis and pancreatic neuroendocrine tumor. This patient was subjected to a genetic test with 80 cancer predisposing genes. The genetic panel revealed the presence of a large pathogenic deletion in the *TSC2* gene, covering exons 2 to 16 and including the initiation codon. No changes were identified in the colorectal cancer and colorectal polyposis genes.

**Discussion and conclusions:**

We describe a case of TSC that presented tumors of the gastro intestinal tract that are commonly unrelated to the disease. The patient described here emphasizes the importance of considering polyposis of the gastrointestinal tract and low grade neuroendocrine tumor as part of the TSC syndromic phenotype.

## Background

Tuberous Sclerosis Complex (TSC) is a genetic disorder with multiorgan involvement, a broad phenotype with inter and intra-familiar variability and well-established clinical diagnostic criteria (Table 1) [1–4]. The incidence of TSC is approximately 1 in 6000–10,000 live births, and in Europe its prevalence has been estimated to be 8.8/100,000 [5]. Germline pathogenic variants in *TSC1* and *TSC2* are identified in 75–90% of patients with the clinical diagnosis and at least 60% of TSC patients do not have a family history of the disease and are considered sporadic [6].

**Table 1:** Criteria for the clinical diagnosis of TSC [1]

Criteria	Description	Observed in the proband
Major	Facial angiofibroma	✓
	Ungueal/peri-ungueal fibroma	
	Hypomelanotic macules	
	Subependymal nodules	✓
	Cortical tubers	✓
	Subependymal giant cell astrocitoma (SEGA)	
	Multiple nodular retinal hamartomas	
	Cardiac rhabdomyoma	
	Renal angiomyolipoma	✓
	Lynphangiomyomatosis	
Minor	Multiple dental enamel macules	
	Rectal polyps	✓
	Osseous cysts	
	Abnormal migration tracts of the White matter	
	Gengival fibromas	
	Non-renal hamartomas	
	Multiple renal cysts	
	“Confetti” skin lesions	

Definitive TSC: Two major criteria or one major and two minor criteria;. Probable TSC: One major and one minor criterion; Possible TSC: One major and two minor criteria

In this report, we describe a patient with the clinical and molecular diagnosis of TSC presenting with two rare associated findings: gastrointestinal polyposis and a pancreatic neuroendocrine tumor. A review of the literature on the subject is provided.

## Case presentation

The patient, a 45-year-old male, was referred for genetic assessment due to clinical findings suggestive of Tuberous Sclerosis Complex (TSC) and polyposis of the gastrointestinal tract. Past medical history included symptoms such as significant seizures since infancy, mild cognitive impairment and adult-onset psychiatric symptoms. These symptoms prompted investigation with a brain magnetic resonance imaging (MRI), which showed subependymal nodules and cortical tubers, two major diagnostic criteria of TSC. Physical examination revealed facial angiofibroma but no additional cutaneous abnormalities were observed. Ophthalmologic, cardiac e pulmonary evaluations did not reveal presence of retinal hamartomas, cardiac rhabdomyomas or pulmonary lymphangioleiomyomatosis. Abdominal computed tomography (CT) scans showed an expansive lesion with heterogeneous enhancement, located in the lower pole of the right kidney, measuring 5.5 cm × 4.0 cm which was later confirmed as a renal angiomyolipoma, another classical sign of TSC. Multiple nodular lesions with arterial enhancement were identified in the liver, the largest one measuring 7.0 × 5.0 cm with features suggestive of secondary implants of unknown origin. In addition, abdominal imaging also showed an expansive lesion in the pancreatic body, with heterogenous enhancement, involving the splenic artery and measuring approximately 6.0 × 4.0 cm. In addition, the patient also had a long history of diarrhea and underwent colonoscopy and upper gastrointestinal endoscopy, revealing presence of more than 50 gastric, colonic and rectal polypoid formations (2 mm to 5 mm).

Family history of cancer was significant for presence of 2 relatives with central nervous system tumors (father and brother diagnosed at ages 62 and 57 years, respectively). Eight additional cancer unaffected siblings were reported. There was also no report of any other family member with clinical features of Tuberous Sclerosis Complex or other genetic conditions. Considering the clinical features of TSC and polyposis of the digestive tract, germline genetic testing was proposed with a next generation sequencing panel validated for large rearrangement screening including 80 cancer predisposition genes in a commercial laboratory. Genes in the panel included: *ALK, APC, ATM, AXIN2, BAP1, BARD1, BLM, BMPR1A, BRCA1, BRCA2, BRIP1, CASR, CDC73, CDH1, CDK4, CDKN1B, CDKN1C, CDKN2A (p14ARF), CDKN2A (p16INK4a), CEBPA, CHEK2, DICER1, DIS3L2, EPCAM, FH, FLCN, GATA2, GPC3, GREM1, HRAS, KIT, MAX, MEN1, MET, MLH1, MSH2, MSH6, MUTYH, NBN, NF1, NF2, PALB2, PDGFRA, PHOX2B, PMS2, POLD1, POLE, POT1, PRKAR1A, PTCH1, PTEN, RAD50, RAD51C, RAD51D, RB1, RECQL4, RET, RUNX1, SDHAF2, SDHB, SDHC, SDHD, SMAD4, SMARCA4, SMARCB1, SMARCE1, STK11, SUFU, TERC, TERT, TMEM127, TP53, TSC1, TSC2, VHL, WRN, WT1* genes. The patient died due to complications of the disease a few months after genetic evaluation. Informed consent to publish this case report was obtained *post-mortem* from his spouse.

**Fig. 1:**
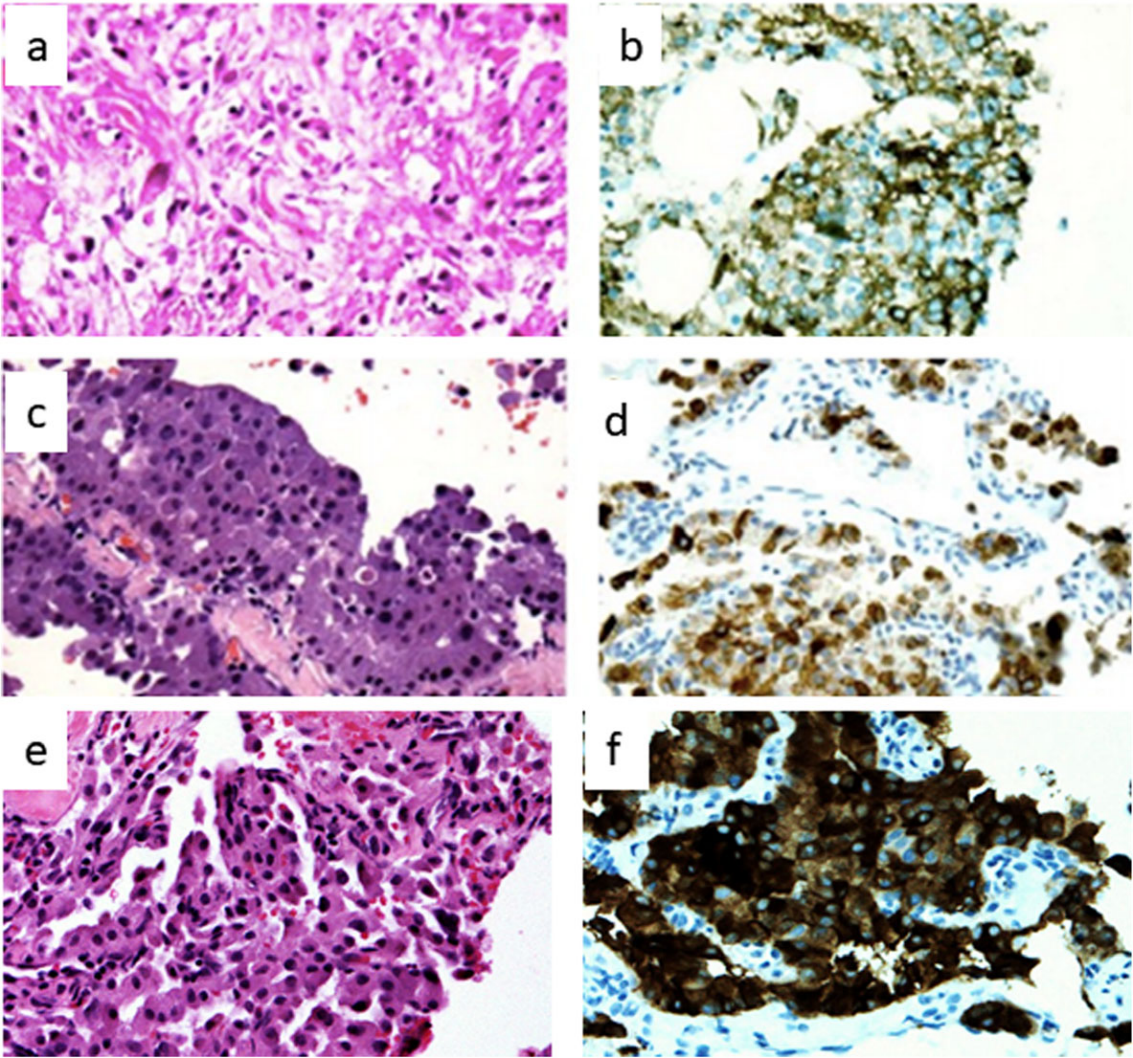
Histologic and immunohistochemistry analyses of the renal and hepatic lesions identified in the proband. **a** and **b**: Kidney biopsy: round cell neoplasia of the kidney (renal angiomyolipoma). **a**) HE: haematoxylin and eosin stain, **b**) Melan A: melanoma antigen. **c**, **d**, **e** and **f**: Liver biopsy (low grade endocrine neoplasia). **c**) HE: haematoxylin and eosin stain, **d**) CGA: chromogranin A antigen, **e**) HE: haematoxylin and eosin stain, **f**) Sinapto: synaptophysin (HE and IHC, 200x)

**Fig. 2:**
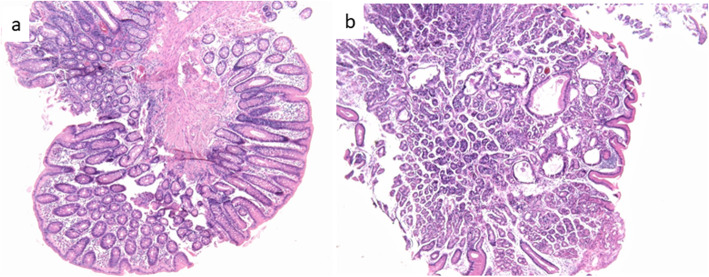
Tubular adenoma and Fundic gland polyp (HE). Panel a: tubular adenomas. Panel b: fundic gland polyps (HE, 200x)

Regarding pathology of the tumors, the haematoxylin and eosin stain (HE) performed in lesion of the right kidney revealed round cell renal tumor with typical morphology (Fig. 1a). The liver lesions were biopsied, showing a histologic pattern suggestive of a low-grade neuroendocrine tumor (NET) (Fig. 1c and e). Biopsies of the pancreatic lesion diagnosed a low-grade neuroendocrine pancreatic tumor (PanNET). Based on the major phenotypic criteria identified in the patient, the clinical diagnosis of TSC with a rare manifestation (PanNET) was established. Partial polypectomies were performed resecting three polyps from the gastric body, two polyps from the right colon and four polyps from the rectum. Histologic examinations of the gastric and colonic/rectal polyps revealed fundic gland polyps and tubular adenomas with low-grade dysplasia, respectively (Fig. 2). Immunohistochemistry (IHC) was performed in the biopsy of the right kidney lesion and demonstrated positive expression of melanoma antigen (Melan A) (Fig. 1b), melanosomal glycoprotein gp100 antigen (HMB45) and smooth muscle actin antigen. The lesions in the liver were confirmed by IHC, showing positivity for multiple citokeratins antigens (40, 48, 50 e 50,6 kDa), chromogranin A antigen (CGA) (Fig. 1d), and synaptophysin (Sinapto) (Fig. 1f).

Germline genetic testing revealed presence of a large pathogenic deletion in *TSC2* gene encompassing exons 2 to 16 and including the initiation codon. No alterations in colorectal cancer/colorectal polyposis genes (*APC, AXINS2, BMPR1A, CDH1, CHEK2, EPCAM, GREM1, MLH1, MSH2, MSH3, MSH6, MUTYH, NTHL1, PMS2, POLD1, POLE, POLE, PTEN, SMAD4, STK11, TP53*) were identified.

## Discussion and conclusions

TSC is an autosomal dominant disease associated with cancer predisposition and multisystemic involvement mainly due to hyperactivation of the mTOR pathway, secondary to loss of function mutations in *TSC1* and *TSC2* [7]. Approximately 15% of the pathogenic variants identified in *TSC2* and 8% of those identified in *TSC1* are large gene rearrangements (LGR) [8], and therefore, genotyping using a methodology that allows LGR detection is important in a diagnostic workup. Although criteria for clinical diagnosis of TSC are well established, expressivity is highly variable, even within families with multiple carriers of the same pathogenic variant and simplex cases with de novo mutations are not uncommon reaching up to 86% in some cohorts [9]. The recent, increased access to multigene panel testing to investigate suspected hereditary cancer has resulted in molecular diagnosis of individuals without the classic clinical criteria or apparently “sporadic” tumors or isolated clinical features of the disease.

In this report, we describe a patient fulfilling criteria for the clinical diagnosis of TSC, such as cortical tubers, facial angiofibroma and renal angiomyolipoma (Table 1 and Fig. 1a and b) carrying a previously described large *TSC2* rearrangement with two uncommon clinical manifestations of the disease: gastrointestinal adenomatous polyposis and a metastatic pancreatic neuroendocrine tumor. The occurrence of numerous colonic and rectal polyps, characterized in this patient as tubular adenomas, is a symptom associated with gastrointestinal polyposis and colorectal cancer syndromes, such as Familial adenomatous polyposis (FAP), a rare autosomal, dominant hereditary disease [10]. FAP is caused by a germline mutation in the *APC* gene [11]. Besides FAP, other syndromes could be associated, including mismatch repair deficiency (biallelic *MLH1*, *MSH2*, *MSH6*, *PMS2* gene mutations), polymerase proofreading-associated polyposis (*POLD1*, *POLE* genes), juvenile polyposis (*SMAD4*, *BMPR1A* genes) and *MUTYH*-associated polyposis [12]. Sequence changes and exonic deletions/duplications were evaluated in all of these associated genes and negative results exclude these syndromes in this patient.

The heterozygous *TSC2* exon 2–16 deletion identified is also known as deletion of exons 1–15 in the literature. Truncating variants including gross deletions in *TSC2* are known to be pathogenic. The 5′ end of the deletion remained undetermined as it was beyond the assayed region and the 3′ boundary was probably within intron 16 of the *TSC2* gene. This deletion is expected to result in complete removal of the TSC1 binding domain (T1BD) the N-terminus of the TSC2 protein in one of the alleles. This domain is critical for TSC1-TSC2 interaction (formation of TSC complex) and abnormal or absent TSC complex results in TSC2 ubiquitination and degradation. This in turn eliminates inhibition of the conversion of Rheb-GTP which accumulates and directly activates the mTORC1 pathway [13, 14].

Three previous reports describe TSC patients carrying the same germline *TSC2* exon 2–16 (a.k.a. exon 1–15) deletion [15–17]. However, according to the information available, none of them presented the uncommon clinical features reported here (neuroendocrine tumors or gastrointestinal polyps) (Tables 2 and 3); although it is possible that due to their ages, these phenotypes would not yet be identifiable. Interestingly, Mortaji et al., 2018 described an adult TSC patient who presented both, a pancreatic NET and gastrointestinal (GI) polyps. But different from the case presented here, they were hamartomatous/inflammatory polyps [40]. Although rectal polyps are included as a minor clinical diagnostic criterion for TSC, there is no mention to polyps in other portions of the GI tract and the vast majority of polyps described in TSC patients are hamartomatous [19, 43]. Gastric fundic polyps (FGPs) are considered hamartomas and tuberin protein (codified by *TSC2* gene) seems to play an important role in pathogenesis of sporadic FGPs by deregulation of cell proliferation. The altered cellular localization of tuberin interrupts its interaction with hamartin protein (codified by *TSC1*) preventing the formation of TSC complex that regulates mTORC1 pathway, responsible for cell proliferation and protein synthesis signaling pathways. In addition, altered cellular localization of tuberin may preclude its negative regulation of gene transcription mediated by tuberin-associated proteins glucocorticoid receptor (GCR) [44]. We identified only one case report of an adolescent TSC patient with tubular adenomatous polyps of the GI tract. The report by Digoy et al. (2000), and the case reported here, presented with a high number of GI tract polyps (unlikely somatic in origin) and a negative comprehensive evaluation of known polyposis genes, reinforce that GI polyposis with different histologies is likely part of the TSC phenotype and should be considered in the differential diagnosis [18]. Of note, glandular fundic polyps and tubular adenomatous polyps could be two different expressions of the same germline variation.

**Table 2:** Previous reports of GI tract polyposis in TSC patients

Reference	Age	TSC features	GI tract alterations	Mutant gene^a^
[18]	17 yo female	Mental retardation, brain astrocytoma, facial angiofibroma, hypomelanotic macules, renal angiomyolipoma	Rectal adenocarcinoma and multiple (> 50) tubular adenomas	NA
[19]	42 yo female	Seizures, renal and liver angiofibromas, multiple subependymal calcifications of the brain, lymphangioleiomyomatosis of the lungs, cerebromalacia	Multiple gastric (fundic) hamartomas	NA
[20]	51 yo female	Epilepsy, mild cognitive impairment, ungueal fibromas.	More than 50 sessile polyps of small size scattered through the left colon and rectum	*TSC1* c.1257delC (p.Arg420Glyfs*20)

^a^*NA* Not assessed

**Table 3:** Previous reports of neuroendocrine tumors in individuals with a clinical or clinical and molecular diagnosis of TSC

Reference	Summary	Mutant gene^a^
	**Pituitary NET**	
[21]	Case report: 12 yo male with a GH-oma and acromegalic gigantismo.	NA
[22]	Case report: 25 yo female with hyperprolactinaemia, amenorrhoea and galactorrhoea after delivery of 3rd child.	NA
[23]	Case report: 32 yo male with an ACTH-oma and Cushingoid features.	NA
[24]	Case report: 13.5 yo male with an ACTH-oma, short stature, abnormal distribution of fat tissue and rounded face, plethora and acne.	NA
	**Parathyroid NET**	
[25]	Case report: 20 yo female with parathyroid hyperplasia, and on autopsy multiple endocrine adenomatosis affecting, in addition to the parathyroid, the pituitary (a non-functioning pituitary adenoma), adrenals and pancreas (islet cell tumour).	NA
[26]	Case report: 14 yo female with a parathyroid adenoma, anorexia, occasional nausea and vomiting, polydipsia, polyuria, constipation and generalised osteoporosis	NA
[27]	Case report: 15 yo male with a parathyroid adenoma and acute pancreatitis	NA
	**Rectal NET**	
[28]	Case report: 18 yo female with Proteus syndrome and TSC, subcortical tubers, developmental delay, seizure disorder, bilateral renal angiomyolipomas, ventricular rhabdomyomas, choledochal cyst, epidermal inclusion cysts, skin tags, synchronous well-differentiated L-cell rectal neuroendocrine tumor and leiomyomatosis-like lymphangioleiomyomatosis of the rectum.	*TSC2*
	**Pancreatic NET**	
[29]	Case report: 24 yo female with insulinoma and symptomatic hypoglycaemia and novel onset of seizures	NA
[30]	Case report: 23 yo male with insulinoma and recurrent seizures presented after 15 years of being seizure free	NA
[31]	Case report: 34 yo male with a pancreatic gastrinoma, presenting with reflux esophagitis and massive weight loss	NA
[32]	Case report: 28 yo male with insulinoma and behavioral changes characterised by episodes of agitation and, at other times, lethargy	NA
[33]	Case report: 18 yo female with insulinoma with symptomatic hypoglycaemia.	NA
[34]	Case report: 12 yo male with a malignant islet cell tumour	*TSC2* (nonsense)
[35]	Case report: 43 yo male with insulinoma and episodes of Episodes of sweating and dizziness.	NA
[36]	Case report: 6 yo male with a malignant islet cell tumour of pancreas	*TSC2* (nonsense)
[37]	Case report: 39 yo male with a pancreatic islet cell tumor and lichenified hyperpigmented plagues (paraneoplastic process)	*TSC2* (1 bp ins)
[38]	Case report: 31 yo male with TSC, multiple congenital subependymal nodules, bilateral cortical tubers, seizures and a malignant (metastatic) pancreatic neuroendocrine tumor.	NA
[39]	Description of 5 patients with TSC (clinical diagnosis) and pancreatic tumors, 2 of them confirmed pancreatic neuroendocrine tumors, localized in the pancreatic tail (5 yo male with a 26 mm lesion and 12 yo male with a 10 mm lesion).	NA
[40]	Case report: 35 yo female with TSC, adenoma sebaceum, shagreen patch and hypopigmented macules, bilateral renal angiomyolipomas and Hurthle cell adenoma. Multiple benign hamartomatous and inflammatory-type polyps in the cecum, sigmoid colon, and rectum. Pancreatic well-differentiated neuroendocrine tumor.	*TSC1* (2 bp del)
	**Pheochromocytoma**	
[41]	Case report: 29 yo female with a pleomorphic adrenal pheochromocytoma, recurrent fever and abdominal pain. Abdominal recurrence involving the spinal cord	NA
	**Carcinoid tumor**	
[42]	Case report: 34 yo female with renal cysts and a bronchial carcinoid presenting by hemoptysis 2 years after diagnosis of “sporadic” lymphangiomyomatosis (LAM). On post-mortem examination LAM was observed in the lungs, mediastinal lymph nodes, kidneys and uterus. LOH for the *TSC1* mutation observed in several tissues but not in the carcinoid tumor.	*TSC1* (nonsense)

^a^*NA* Not assessed

Finally, pancreatic neuroendocrine tumors (PanNET) are most commonly sporadic but have been reported previously in association with TSC and in other inherited cancer syndromes such as von Hippel-Lindau disease, Neurofibromatosis type 1 and Multiple endocrine neoplasia type 1 [45]. Most TSC patients diagnosed with NETs have pancreatic NETs, but NETs in other organs must be considered as part of the TSC phenotype. Recent studies have shown that most TSC patients with Pancreatic NETs have a germline pathogenic variant in *TSC2* gene, as observed in our case. The multiple reports of NET in TSC patients and recent evidence for a pivotal role of TSC1 and TSC2 proteins in NET development and tumor’s response to mTORC1 modulating interventions, point to a direct relationship between loss of function variants in *TSC1* and *TSC2* and NET suggesting that TSC clinical criteria should be modified to include NETs [46–48]. To our knowledge, there are no previous reports of tubular adenomatous polyposis in multiple segments of the GI tract in carriers of *TSC2* germline pathogenic variants (Table 3). In a previous report describing molecular features of TSC patients, none of the probands reported GI tract polyposis [49].

In conclusion, there is currently no recommendation for GI polyp or PanNET screening, probably given the rarity of these findings, in TSC patients. Gastric and colorectal polyps and PanNETs are also not considered as phenotypic criteria for the clinical diagnosis of the syndrome. The patient described here, with confirmed molecular diagnosis of TSC underscores the importance of considering GI tract polyposis and NETs as part of the syndromic phenotype.

## Data Availability

Not applicable.

## Abbreviations

TSC: Tuberous Sclerosis Complex
MRI: Magnetic resonance imaging
CT: Computed tomography
HE: Hematoxylin and eosin
NET: Neuroendocrine tumor
PanNET: Neuroendocrine pancreatic tumor
IHC: Immunohistochemistry
Melan A: Melanoma antigen
HBM45: Melanosomal glycoprotein gp100 antigen
CGA: Chromogranin A antigen
Sinapto: Synaptophysin
LGR: Large gene rearrangements
FAP: Familial adenomatous polyposis
T1BD: TSC1 binding domain
GI: Gastrointestinal
FGPs: Gastric fundic polyps
GCR: Glucocorticoid receptor
